# Virtual Reality Rehabilitation’s Impact on Negative Symptoms and Psychosocial Rehabilitation in Schizophrenia Spectrum Disorder: A Systematic Review

**DOI:** 10.3390/healthcare9111429

**Published:** 2021-10-23

**Authors:** André Novo, Jéssica Fonsêca, Bárbara Barroso, Manuel Guimarães, Afonso Louro, Hélder Fernandes, Rui Pedro Lopes, Paulo Leitão

**Affiliations:** 1 Campus de Santa Apolónia, Instituto Politécnico de Bragança, 5300-253 Bragança, Portugal; jessica.fonseca@ipb.pt (J.F.); bbarroso@ipb.pt (B.B.); helder@ipb.pt (H.F.); rlopes@ipb.pt (R.P.L.); pleitao@ipb.pt (P.L.); 2 Center for Health Technology and Services Research (CINTESIS), R. Plácido da Costa, 4200-450 Porto, Portugal; 3 Campus de Santa Apolónia, Research Center in Digitalization and Intelligent Robotics (CeDRI), 5300-253 Bragança, Portugal; manuel.guimaraes@ulsne.min-saude.pt; 4 Avenida Abade de Baçal, Mental Health Department, Unidade Local de Saúde do Nordeste, 5301-852 Bragança, Portugal; aroso.louro@ulsne.min-saude.pt; 5 Rua José Alberto Reis, Health Sciences Research Unit: Nursing (UICISA), 3000-232 Coimbra, Portugal

**Keywords:** virtual reality, virtual reality exposure therapy, serious game, schizophrenia

## Abstract

Background: Schizophrenia is a chronic psychiatric disorder with symptoms that can severely affect patients’ quality of life. While drug administration inhibits most of the positive symptoms, it fails to effectively treat the negative symptoms and in turn relies on rehabilitation therapies. With technological progress, virtual reality (VR) has been the target of recent studies in terms of mental health rehabilitation and has been shown to be a tool with ecological validity in order to introduce the individual to situations similar to their daily life. Methods: From July to August 2021, we conducted a systematic review with the purpose of understanding the impact of virtual reality rehabilitation on negative symptoms and psychosocial rehabilitation in the schizophrenia spectrum. The searches were performed in the databases Web of Science, Scopus, and PubMed. Results: In our search, we identified 201 results, where 108 duplicates were excluded, resulting in a final balance of 93. After reading and assessing the titles and abstracts, 66 studies were excluded. Of the remaining 27 reports, 23 were excluded for not meeting the previously defined eligibility criteria, resulting in the inclusion of four studies in this systematic review. Conclusions: The available data on the specific topic was limited and could have been more complete. However, in our review, we were able to identify statistically relevant results demonstrating the effectiveness of intervention. We could find medium to large effects, allowing VR to be an ally for rehabilitation of symptoms related to schizophrenia.

## 1. Introduction

Schizophrenia affects around 1% of the world’s population. According to the WHO, the psychiatric illness affects around 20 million people worldwide and is thus the most common psychotic illness [1]. It is characterized by delusions, hallucinations, anhedonia, and apathy [2].

Social impairment is a core feature of schizophrenia, which presents a major barrier towards recovery [3]. People with schizophrenia may experience significant difficulties in social cognitive functioning, including problems understanding actions, emotions and intentions, social perception, empathy, mental state attributions, and theory of mind, which lead to poor functional outcome in this disorder [2,4].

Some of the psychotic symptoms, such as hallucinations and delusions, are partially improved by antipsychotic medication, but the path to recovery is hampered by social impairments [3,4].

In general, antipsychotics do not appear to have good efficacy on negative symptoms [5].

Negative symptoms are associated with detrimental effects on patients’ functional status, quality of life, and long-term outcome and are among the most important unmet needs in this disorder [4,6]. Their clinical expression is less evident than that of positive symptoms because they may be masked by positive symptoms and may coexist with or be confused with affective symptoms or cognitive impairment [6]. In this sense, rehabilitation is an essential component of universal health coverage (https://www.who.int/health-topics/rehabilitation#tab=tab_1, accessed on 1 September 2021) and can be defined as interventions needed when someone faces limitations in performing their daily activities [7]. Mental health rehabilitation is a dynamic evidence-based model that provides comprehensive and continuous care plans focused on the person with severe and persistent psychiatric illness. It is closely linked to community integration and increased quality of life [8].

Considering the therapeutic deficiency, researchers are increasingly engaged in the study of new technologies for application in medicine.

The concept of virtual reality (VR) was introduced in the 1950s and is defined as a computer-generated simulation, such as a set of images and sounds representing a real place or situation, that can be interacted with, in an apparently real or physical way, by a person using special electronic equipment [9].

In psychiatry in particular, traditional treatment tools have been limited mainly to psychotherapy and medication. Rehabilitation supported by digital technologies has thus emerged as a solution to support health professionals by providing high-intensity, repetitive, and task-specific exercises to improve the rehabilitation process [10]. VR has already been applied to the treatment of various pathological conditions. Several clinical studies have demonstrated its effectiveness in the treatment of post-traumatic stress disorder, anxiety disorder, and specific phobias [9,11]. It has also been recognized as a promising tool for the assessment and treatment of mental disorders [12]. In patients with positive symptoms, the exercise of playing on the internet contributes to the reduction of symptoms, such as hallucinations or others [13]. Some studies have started to apply VR tools to try and contrast the cognitive decline of patients over time, particularly in neurodegenerative diseases, such as Alzheimer’s disease, frontotemporal dementia, Parkinson’s disease, and multiple sclerosis [14]. Moreover, the possibility to play with more than one person also contributes to improving social interaction [15].

VR provides simulating environments where patients can explore and experience actions that present a significant degree of difficulty in their lives, such as going shopping, memorizing the way to the supermarket, or being in a certain place at a certain time [16].

VR has also been suggested as a rehabilitation treatment with great ecological advantage, presenting itself as a promising and safe instrument capable of reproducing daily situations in a virtual environment and allowing the user to have an experience as close as possible to real life, thereby overcoming the barrier of accessibility. Other relevant advantages can also be perceived, such as allowing errors and repetitions in a more dynamic way, thus helping users to develop self-confidence and motivation to safely achieve the goal.

For the health team, the lack of human resources can hinder adequate rehabilitation. This technique overcomes this problem. It is also possible to have more concrete feedback on the patient’s performance with the collection and cross-checking of data in a more effective way. This allows the adequacy of treatment as well as difficulties to be identified.

Our aim in this review was to analyze the outcomes of interventions in order to understand the impacts of VR rehabilitation therapy on negative and psychosocial symptoms in patients diagnosed with schizophrenia.

## 2. Materials and Methods

### 2.1. Search Strategy

This is a systemic review that follows the PRISMA (Preferred Reporting Items for Systematic Reviews and Meta-Analysis) guidelines [17]. It has been registered in the international database, PROSPERO, under number CRD42021266466.

The reviewers J.F. and A.N. started searching the Web of Science, PubMed, and Scopus databases on 07/07/2021. The following terms were used: ((Virtual Reality) OR (Virtual Reality Exposure Therapy) OR (Serious Games)) AND (Schizophrenia).

### 2.2. Eligibility Criteria

There were no restrictions as to date of publication. However, in relation to languages, only English, Spanish, or Portuguese were selected. All studies had to meet the following eligibility criteria.

1. Participants: Patients diagnosed with schizophrenia
2. Intervention: Virtual reality exposure therapy
3. Comparison: No intervention with virtual reality
4. Outcomes to be considered: Negative symptoms and psychosocial functioning
5. Study design: Randomized controlled trial

### 2.3. Data Extraction and Synthesis

The synthesis was conducted in the following steps:

(1) Identification: Records were identified through database search and reference screening. All references were exported to the data management software (Rayyan) [18], and all duplicates were removed. After this step, the data were exported to the Mendeley reference manager.
(2) Screening: Two reviewers independently examined the titles and abstracts of studies applying the eligibility criteria, and all irrelevant studies were excluded.
(3) Eligibility and selection: Full reading of the most relevant records was conducted, and all papers not meeting the inclusion criteria were excluded from the systematic review. If there were disagreements, these were resolved by a third reviewer.
(4) Data extraction: Reviewers J.F. and A.N. sought to extract the following data: title, year, authors, study design, sample size, intervention type, DSM, frequency and duration of interventions, purpose of study and assessment methods (Table 1), technology used, intervention outcomes, other results, and conclusion. Pre- and postintervention outcomes were analyzed and measured using specific scales used in each study.

### 2.4. Risk of Bias Assessment

The risk of bias for the included studies was assessed with Cochrane risk of bias tool for randomized trials, version 7 (RoB 2) [19].

## 3. Results

### 3.1. Characteristics of the Included Studies

**Figure 1:**
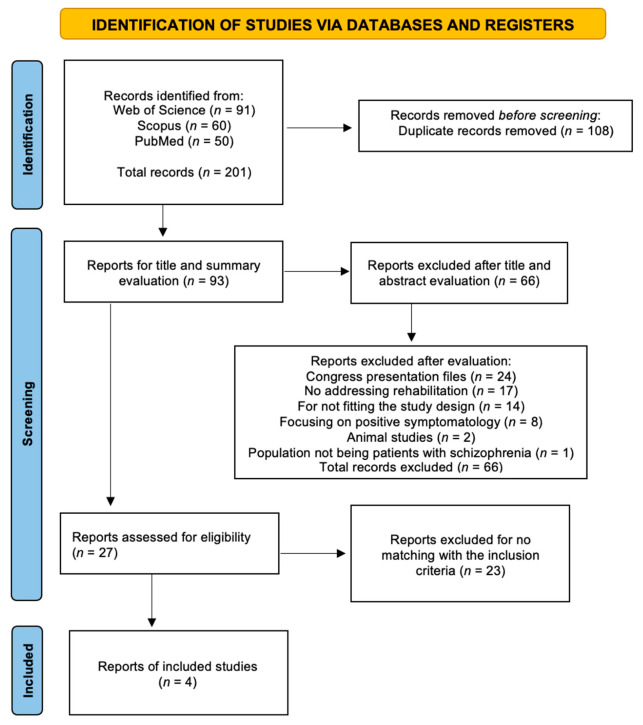
PRISMA flow diagram highlighting the selection process for the studies included in this systematic review [17].

The database search yielded 201 results, 91 in Web of Science, 60 in Scopus, and 50 in PubMed. A total of 108 duplicates were excluded, resulting in a final balance of 93. After reading and evaluating the titles and abstracts, 66 studies were excluded: 24 because they were congress presentation files, 17 because they did not address rehabilitation, 14 because they did not fit the previously defined study design, 8 because they focused on positive symptomatology, 2 because they were animal studies, and 1 because it did not involve patients with schizophrenia. This resulted in 27 studies for the application of the previously defined eligibility criteria. After exclusion of 23 studies for not meeting the criteria, a total of four studies were finally included (Figure 1).

### 3.2. Purpose of Study, Procedures, and Assessment Methods

#### 3.2.1. Purpose and Procedures

Vass et al. (2020) aimed to develop a novel virtual reality (VR)-based targeted theory of mind (ToM) intervention (VR-ToMIS) specifically designed for patients with schizophrenia (VR-ToMIS = VR-based ToM Intervention in Schizophrenia) (Table 2) [20]. The ToM intervention is based on the theory of mind applied in VR. The term theory of mind is used to assess an individual’s degree of ability to empathize and understand others. To do this, Vass and his team conducted eight simulation-based virtual sessions and an extra initial session designed to help patients understand the method. The active sessions were based on three consecutive steps, preceded by a brief warm-up that provided sufficient time to review the intensity of activity between sessions (homework and behavior change monitoring). Key change procedures, such as “how to hold a conversation” were revisited [20].

Park et al. (2011) sought to examine the utility of virtual reality (VR) in social rehabilitation and its role in skill formation. This study was designed to compare social skill formation (SST) using VR staging (SST-VR) with traditional staging (SST-TR) with the aim of finding advantages of using VR in the social rehabilitation of patients with schizophrenia. The hypothesis was that it could improve training outcomes by increasing participant motivation [21].

The sessions consisted of three consecutive trainings: five conversational skills training sessions (“introduce yourself”, “find a common concern and list the other person”, “start a conversation”, “keep a conversation going”, and “end a conversation”), three assertiveness skills training sessions (“make a demand”, “reject a demand from another person”, and “make a compromise”), and two emotional expression skills training sessions (“express positive emotions” and “express negative emotions”) [21]. Homework from the previous session was reviewed at the beginning of the next session as in Vass et al. (2020). Each session included a therapist model followed by a role-play by the participant and then positive and corrective feedback from the therapists. After identifying deficient skills, the participant was again involved in another role-play of the same scene and also received feedback [21]. Each session included three role-plays with different scenes per participant. The team of López-Martín et al. (2016) aimed to evaluate the effectiveness of using Nintendo Wii^®^ as a therapeutic tool to improve the cognitive domains, self-esteem, and quality of life of patients with schizophrenia [23]. All received the pretreatment measures and the conventional treatment. The experimental group also received 10 sessions of 50 min each of individual therapy using virtual reality twice a week for five weeks [23]. The prominence of the sessions was not described.

Tsang and Man’s (2013) study adopted theory-driven training strategies, and one of the training programmers was enhanced using virtual reality (VR) as an intervention tool. It sought to understand the effectiveness of a new VR-based vocational training system (VRVTS) by evaluating the cognitive performance of the groups [22]. Training tasks included packing, typing, and cleaning tasks. All participants attended at least 3 h of prevocational training. In the VR-based vocational training group (VRG), in addition to prevocational training, participants were also exposed to VR-based vocational training in a virtual boutique environment (VRVTS), whereas in the therapist-administered group (TAG), participants attended a prevention and therapist vocational training program administered in a boutique environment [22]. The content of the VRG and TAG training was the same, but the mode of training differed.

#### 3.2.2. Assessment

Regarding psychopathology, cognitive, and functional assessments, there was no standardized use; each team defined their assessment scales based on their previous studies (Table 3). Vass et al. (2020) worked on pre- and postintervention assessment to assess psychopathology by the Positive and Negative Syndrome Scale (PANSS), but the scoring method was modified [20].

Neurocognitive deficits, on the other hand, were assessed by Repeated Battery for the Assessment of Neuropsychological Status (RBANS) and Wisconsin Card Rating Test (WCST-64). To overcome the complexity of theory of mind (ToM), Baron-Cohen Mind in the Eyes Test (BCMET), the faux pas test, and the cartoon story task were administered to test mental state ability [20].

Park et al. (2011) also assessed symptom severities before and after SST using the Positive and Negative Syndrome Scale (PANSS) being applied by an experienced psychiatrist using. For voice, nonverbal and conversational skills were assessed using 29 items from the Trower Social Behavior Scale (SBS) [21]. For secondary outcomes, they used the Rathus Assertiveness Schedule (RAS) to assess assertiveness on a six-point Likert scale, Relationship Change Scale (RCS) to measure interpersonal relationship skills, and short version of the Social Problem-Solving Inventory—Revised (SPSI-R) to measure the individual’s cognitive, affective, or behavioral responses to real-life problem-solving situations [21].

Tsang and Man (2013) assessed global cognitive functioning using the Brief Neuropsychological Cognitive Examination (BNCE) for specific cognitive functioning. Attention was measured by the Digit Vigilance Test (DVT), memory by the Rivermead Behavioral Memory Test (RBMT), executive functioning by the Wisconsin Card Sorting Test (WCST), and cognitive functioning in the workplace by the Vocational Cognitive Rating Scale (VCRS). The subjects’ own knowledge, skills, and self-efficacy in performing sales-related activities were measured by checklists developed for this project [22].

The work by López-Martín et al. (2016) was the only one to use a single assessment scale.

They used the MATRICS Consensus Cognitive Battery (Spanish version) to measure therapeutic progress in cognitive domains [23].

### 3.3. Diagnosis

In the studies of Vass et al. (2020) and López-Martín et al. (2016), patients were diagnosed using the Diagnostic and Statistical Manual of Mental Disorders (DSM) version IV-TR. Park et al. (2011) and Tsang and Man (2013) described using DSM-IV. However, Park et al. (2011) specified having used axis 1, while López-Martín et al. (2016) also used International Statistical Classification of Diseases and Related Health Problems (ICD-10) (Table 3).

### 3.4. Technology Used

In the pilot study by Vass et al. (2020), the patients participated in simulated social interactions with an avatar in immersive VR environments (ambient; provided by vTime [https://vtime.net/]) (accessed on 1 September 2021). Samsung’s Gear VR equipment was used, including a head-mounted display (HMD), a Samsung S7 smartphone, and a Samsung Simple Controller [20]. In Park et al. (2011), the VR system included a personal computer to render and provide the virtual environment, a head-mounted display (HMD; Eye Trek FMD 250W, OLYMPUS) to display the virtual environment in a more immersive way, and a position tracker (InterTrax2, InterSense) to follow head direction in real time. The VR role-plays were displayed through two different panels: an HMD and a 120-inch screen [21]. For VR training, Tsang and Man (2013) used a desktop computer to run the program in VR-based training group (VRG). The computer was a Pentium IV 2.40GHz CPU with Windows 2000 or higher. In addition, a joystick, keyboard, mouse, 38″ LCD monitor, and a set of stereo speakers were required as input and output devices [22].

In the randomized study by López-Martín et al. (2016), the only information found was that the virtual reality system included the software used, which was the Big Brain Academy game program and a Nintendo^®^ Wii game console connected to a 40-inch LCD TV for the intervention sessions [23].

### 3.5. Sessions and Samples

The number of sessions was the same in Park et al. (2011), Tsang and Man (2013), and López-Martín et al. (2016). All had 10 sessions and with the same duration of intervention of five weeks, and the frequency was twice a week in all three studies [21,22,23]. In Vass et al. (2020), the VR-ToMIS was conducted for nine weeks [20].

Regarding the duration of the sessions, they varied from 30 to 90 min per session: Tsang and Man (2013) = 30 min, Vass et al. (2020) and López-Martín et al. (2016) = 50 min, and Park et al. (2011) = 90 min [20,21,22,23].

### 3.6. Statistical Results of the Interventions

#### 3.6.1. Negative Symptoms

For negative symptoms, two of the included papers made specific symptom assessment. Vass et al. (2020) and Park et al. (2011) used the PASS scale to assess symptom severity [20,21]. The statistical finding of Vass et al. (2020) was that compared to the VR passive condition, the VR-ToMIS group was associated with significant improvements in negative symptoms on the PANSS score, with a large effect size (ηp² = 0.58) [20]. In the study by Park et al. (2011). there were no differences in positive, negative, and general symptoms on the PANSS in the post-SST evaluation [21].

#### 3.6.2. Cognitive and Functional Outcomes

Vass et al. (2020) reported that with regard to the Wisconsin Card Sorting Test (WCST-64) score, VR-ToMIS was associated with significant improvements in only the number of correct responses. However, a trend towards significance was also shown for the rate of nonperseverative errors. A large effect size was found for each of the mentioned variables (ηp² = 0.22–0.24).

Patients’ performance on the visuospatial and attention subtest of the Repeated Battery for the Assessment of Neuropsychological Status (RBANS) differed significantly between the VR-ToMIS and passive VR conditions, with a medium effect size (φ = 0.32–0.34) in BCMET scores; no significant between-group differences were found [20].

In the study by Park et al. (2011), the SBS scores showed significant outcome group effects in the nonverbal skills (*p* = 0.010, partial η² = 0.101) and time effects on all three outcomes (*p* = 0.001, partial η² = 0. 590 on vocal skills; *p* = 0.001, partial η² = 0.268 on nonverbal skills; *p* = 0.001, partial η² = 0.620 on conversational skills), i.e., the SST-VR group had greater improvement in conversational skills than the SST-TR group but less improvement in nonverbal skills [21]. The RAS scores also showed significant group effects (*p* = 0.040, partial η² = 0.066) and temporal effect on all three outcomes (*p* = 0.001, partial η² = 0.297 on RAS score; *p* = 0.022, partial η² = 0.087 on RCS score; *p* = 0.001, partial η² = 0.920 on SPSI-R score). This showed that the SST-VR group had greater improvement in the RAS score [21].

Tsang and Man (2013) observed that there was a significant group interaction effect over time on DVT time (*p* = 0.03, observed power = 0.09), RBMT (*p* = 0.02, observed power = 0.10), WCST error percentage (*p* = 0.01, observed power = 0.78), and WCST conceptual level response percentage (*p* = 0.01, observed power = 0.79) [22].

No significant differences were found between groups regarding DVT time (*p* = 0.90) or RBMT (*p* = 0.15). A significant difference was found between groups for WCST error percentage (*p* = 0.001) and WCST conceptual level response percentage (*p* = 0.001) [22].

The VR-based training group (VRG) showed better performance than both the therapist-administered group (TAG) (*p* = 0.03) and conventional group (CG) (*p* = 0.001) in WCST error percentage. VRG also showed better performance than both TAG (*p* = 0.01) and CG (*p* = 0.001) on the WCST conceptual level response percentage. However, no significant group interaction effect over time was found for the BNCE [22]. Patients receiving VRVTS were found to show improvements in cognitive functioning [22].

López-Martín et al. (2016) stated that there was a significant increase in the T-score in the experimental group for all domains compared to a slight decrease in the control group. The domains processing speed, attention/vigilance, working memory, and problem-solving showed statistically significant differences in favor of the experimental group (*p* ≤ 0.002) [23]. The magnitudes of the effect can be considered large, with the partial eta squared ranging from 0.23 to 0.32; the figure was highest for working memory. Verbal learning showed a significant increase in favor of the experimental group (*p* = 0.009) with a smaller effect size, although it can also be considered large (partial eta squared = 0.17). Visual learning showed a significant difference in favor of the experimental group, trending towards statistical significance (*p* = 0.064) with a medium effect size (partial eta squared = 0.088) [23].

The results showed that the experimental group achieved clinically relevant improvement in all six cognitive domains compared to the control group. Five of them were statistically significant (*p* < 0.01), with visual learning being the exception [23].

### 3.7. Patient Feedback

According to Tsang and Man’s (2013) feedback, VRVTS was more interesting and useful than conventional training [22]. In the study by Park et al. (2011), although there was no difference between the two groups in the dropout rate, the VRVTS group showed a higher attendance rate than the VR-ToMIS group (*p* = 0.019) [21], showing that the intervention was more motivating.

After the last session, the team of Vass et al. (2020) invited all participants in the VR-ToMIS group to give their subjective opinion about the intervention. According to the patient feedback, the intervention was engaging, interesting, and safe to use [20].

In the study by López-Martín et al. (2016), the researchers felt that the intervention could play a key role in patient motivation and adherence, but there was no data on patient satisfaction [23].

## 4. Discussion

The number of studies available (four) was the major limitation of this review. Moreover, it was clear that there was lack of standardization regarding the scales used, both in cognitive assessment and in the assessment of symptoms. We identified a large number of scales taking into account the number of studies analyzed. Another limitation was that negative symptoms were not assessed in isolation. Of all the studies included in this review, only two assessed the impact of VR therapy on the symptoms presented in schizophrenia spectrum disorder. However, this assessment occurred in a nonextensive manner, suggesting that this is a topic to be explored in future interventions.

Moreover, as previously noted, it is possible to analyze physiological signs of symptoms during exposure to a given environment or social situation reproduced in VR, such as heart rate and blood pressure. Some parameters can be measured directly during the session to map the interactions between controlled virtual social environments in relation to the domains of symptoms, physiological responses, and behavior. This allows a more personalized, contextual, and objective diagnostic assessment, making data cross-checking even more efficient [11].

**Figure 2:**
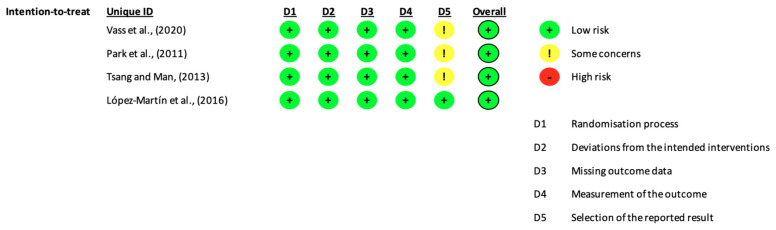
Risk of bias assessment for the included studies [19].

The number of scales used to evaluate the results of each study induced a risk of bias, more specifically in domain 5 of Rob 2. However, our judgment was that the risk was “low” because it is understandable that several scales are used given the cognitive complexity of the patients and the pathology in question (Figure 2).

Regarding the diagnosis, we could identify that some studies, despite the recent publication date, used older version of the Statistical Manual of Mental Disorders (DSM) (Table 3). Park et al. (2011) used edition IV Axis I, while Tsang and Man (2013) used edition IV. Considering the dates of publication, it is reasonable to use these editions. However, Vass et al. (2020) and López-Martín et al. (2016) used version IV-TR, even though the most current edition DSM V has been in force since 2013.

We sought to understand the impact of VR rehabilitation through statistical analysis of the intervention outcomes on negative symptoms and cognitive and functional performance assessments. We found a large effect size in the results of negative symptoms. In cognitive and functional outcomes, better results were also found in the groups that received the intervention with VR. We also observed *p* values < 0.05 for the most part when comparing the groups exposed to intervention with the control groups, reinforcing the significant difference.

Another topic to be discussed is the relatively small sample size to validate intervention. Further studies with a larger number of participants are required.

Regarding the acceptability of the treatment, as cited in Barch (2005), some previous studies have reported that motivation is responsible for the relationship between social functioning and cognition in schizophrenics [24], making satisfaction in performing the treatment one of the most important factors in the rehabilitation of negative symptoms in this pathology. As mentioned earlier in this review, feedback on the interventions was very positive from the groups exposed to VR treatment, which is a positive point when talking about spectrums.

The average number of sessions and the duration of interventions included in this review was 9.75 sessions and 55 min, respectively.

As for the sample size, correlating the studies, the average was 21 participants for the controls and 21.5 for the experimental group in VR.

Based on this review, the most appropriate number of sessions would be 55 min with a minimum duration of 10 sessions.

Virtual games as an auxiliary tool for rehabilitation can be a very interesting path for the future to benefit people diagnosed on the schizophrenia spectrum [10].

## 5. Conclusions

According to analysis, there were limited studies available that fit our selection criteria aimed at examining the impact of VR rehabilitation on negative and psychosocial symptoms in schizophrenia. Nevertheless, we found that VR therapy has a statistically significant impact on the treatment of negative and psychosocial symptoms. As shown in this analysis, there were medium to large effects. However, it is possible to increase future interventions to broaden the way patients are assessed. Vital signs can be monitored and the subtopics of negative symptom assessment can be explored in a more specific way. As a suggestion, the seven domains assessed in the PANSS can be used for specific assessment. In order to try to fill the gaps found in the form of assessment, this research team will continue in the future by analysing in more detail the seven domains assessed in the PANSS.

## Figures and Tables

**Table 1:** Classification of assessment scales.

Scale	Brief Description
Scale	Brief description
PANSS	Positive and Negative Syndrome Scale
WCST-64	Wisconsin Card Sorting Test
BCMET	Baron-Cohen Mind in the Eyes Test
RBANS	Repeated Battery for the Assessment of Neuropsychological Status
SBS	Social Behavior Scales
RAS	Rathus Assertiveness Schedule
RCS	Relationship Change Scale
SPSI-R	Social Problem-Solving Inventory—Revised
BNCE	Brief Neuropsychological Cognitive Examination
DVT	Digit Vigilance Test
RBMT	Rivermead Behavioral Memory Test
WCST-CV4	Wisconsin Card Sorting Test—Computer Version 4
VCRS	Vocational Cognitive Rating Scale
MCCB-MATRICS	MATRICS Consensus Cognitive Battery (MCCB)

**Table 2:** Classification of intervention types.

Intervention Type	Description
VR-ToMIS	Virtual reality (VR)-based targeted theory of mind (ToM). Immersive VR-based targeted ToM intervention that is especially designed for patients with schizophrenia (VR- ToMIS = VR-based ToM Intervention in Schizophrenia) [20]
SST-VR and SST-TR	VR role-playing (SST-VR) and SST using traditional role-playing (SST-TR). The virtual environments are used as simulators of the scenes and avatars as actors in VR role-plays, whereas verbal, writing, picture, and video supplies are used as simulators of the scenes. SST therapists are used as actors in TR role-plays [21].
VRVTS	VR-based vocational training system (VRVTS). Inpatients with schizophrenia are randomly assigned to a VR-based vocational training group (VRG), a therapist-administered group (TAG), and a conventional group (CG) [22].
Big Brain Academy game	A virtual reality system and software using the Nintendo^®^ Wii video console [23].

**Table 3:** Articles included in the review (sorted alphabetically by author).

Title 1	Study Purpose	Study Design	Sample Size	Intervention Type and Duration	DSM	Scales
López-Martín et al. (2016) [21]	To evaluate the effectiveness of a VR-based gaming program for the improvement of cognitive domains in patients with schizophrenia.	RCT	40 patients Control group (*n* = 20) Experimental group (*n* = 20)	Big Brain Academy Game, 5 weeks, 50 min per session	DSM-IV-TR	MCCB-MATRICS
Park et al. (2011) [19]	To compare SST using VR role-playing (SST-VR) to SST using traditional role-playing (SST-TR)	RCT	64 participants SST-VR group (*n* = 32) SST-TR group (*n* = 31)	SST-VR and SST-TR, 5 weeks, 90 min per session	DSM-IV Axis I	SBS RAS RCS SPSI-R PANSS
Tsang et al. (2013) [20]	To investigate the efficacy and effectiveness of VR as a cognitive intervention for enhancing vocational outcomes.	RCT	75 participants VR-based training group (VRG, *n* = 25) Therapist-administered group (TAG, *n* = 25) Conventional group (CG, *n* = 25)	VRVTS, 5 weeks, 90 min per session	DSM-IV	BNCE DVT RBMT WCST-CV4 VCRS
Vass et al. (2020) [18]	To evaluate the feasibility and tolerability of VR-ToMIS	RCT	17 patients VR-ToMIS (*n* = 9) Passive VR (*n* = 8)	VR-ToMIS and Passive VR, 9 weeks, 50 min per session	DSM-IV-TR	PANSS WCST-64 BCMET RBANS

PANSS = Positive and Negative Syndrome Scale; WCST-64 = Wisconsin Card Sorting Test; BCMET = Baron-Cohen Mind in the Eyes Test; RBANS = Repeated Battery for the Assessment of Neuropsychological Status; SBS = Social Behavior Scales; RAS = Rathus Assertiveness Schedule; RCS = Relationship Change Scale; SPSI-R = Social Problem-Solving Inventory—Revised; BNCE = Brief Neuropsychological Cognitive Examination; DVT = Digit Vigilance Test; RBMT = Rivermead Behavioral Memory Test; WCST-CV4 = Wisconsin Card Sorting Test—Computer Version 4; VCRS = Vocational Cognitive Rating Scale; MCCB-MATRICS = MATRICS Consensus Cognitive Battery (MCCB); VR-ToMIS = VR-based ToM Intervention in Schizophrenia; SST = social skills training (SST); SST-VR = social skills training using VR; SST-TR = social skills training using traditional staging.

## Data Availability

Not applicable.
